# Simultaneous suppression of disturbing fields and localization of magnetic markers by means of multipole expansion

**DOI:** 10.1186/1477-044X-2-6

**Published:** 2004-09-01

**Authors:** Bernd Hilgenfeld, Jens Haueisen

**Affiliations:** 1 Biomagnetic Center, Department of Neurology, Friedrich Schiller University Jena, Germany

## Abstract

**Background:**

Magnetically marked capsules serve for the analysis of peristalsis and throughput times within the intestinal tract. Moreover, they can be used for the targeted disposal of drugs. The capsules get localized in time by field measurements with a superconducting quantum interference device (SQUID) magnetometer array. Here it is important to ensure an online localization with high speed and high suppression of disturbing fields. In this article we use multipole expansions for the simultaneous localization and suppression of disturbing fields.

**Methods:**

We expand the measurement data in terms of inner and outer multipoles. Thereby we obtain directly a separation of marker field and outer disturbing fields. From the inner dipoles and quadrupoles we compute the magnetization and position of the capsule. The outer multipoles get eliminated.

**Results:**

The localization goodness has been analyzed depending on the order of the multipoles used and depending on the systems noise level. We found upper limits of the noise level for the usage of certain multipole moments. Given a signal to noise ratio of 40 and utilizing inner dipoles and quadrupoles and outer dipoles, the method enables an accuracy of 5 mm with a speed of 10 localizations per second.

**Conclusion:**

The multipole localization is an effective method and is capable of online-tracking magnetic markers.

## Background

The transport of capsules in the alimentary tract underlies complex influencing factors like the patients peristalsis, the hydration and the form and size of the capsules. A procedure which allows the instantaneous localization of the capsules supports a number of patient examinations as well as examinations of new drug forms [1-4]. Capsules can be marked radioactively (scintigraphy) or magnetically. The scintigraphy [5] has a lower time resolution compared to the magnetic localization, and due to radiation it is not appropriate for examinations with healthy probands.

The localization of magnetically marked capsules (magnetic markers) must be spatially accurate and with high temporal resolution. For the spatial localization the marker field must be separated from the external magnetic disturbing fields. This separation can be achieved by splitting the magnetic field in multipole moments [6]. The method proposed utilizes the multipole moments directly for the determination of the position and the magnetic moment of the marker. Thus, the separation of disturbing fields and the localization are integrated numerically effective into one procedure. This allows a fast online-localization of the marker capsules.

Multipole expansions are used also to model spatially distributed biological sources such as brain currents [7,8].

The application of multipoles for the localization of magnetic dipoles is described in [9,10], and is used in other technical areas without disturbing field suppression [11].

Marking of capsules and pills takes place by partially filling them with black iron oxide (Fe_3_O_4_) which is subsequently magnetized up to saturation. The magnetic field measurement is performed within magnetically shielded rooms by the use of highly sensitive SQUID arrays. For the investigation at hand we conduct simulation runs to determine the performance of the multipole localization.

## Methods

### Algorithm

The field of a magnetic marker located adjacent to the point of origin can be expressed by a multipole expansion in Cartesian coordinates (*x*_1_, *x*_2_, *x*_3_). If the distance  between marker and origin is small compared to the distance  between a magnetic sensor and the origin, the field of the marker at the sensor position is given by the first elements of the multipole expansion. With the notation



follows



with *c*^m ^being the dipole, quadrupole and octopole moments of the field expansion.

The form functions  arise from a Taylor series expansion in the parameter  with  and . It holds



With the Kronecker delta  follows





and



Conversely, a Taylor series expansion of – compared to the sensor coordinates – far away located field sources in the parameter  with  yields a multipole expansion of external disturbing fields:



We denote the multipole moments *c*^ex ^of the expansion of fields of external sources as "outer moments" to distinguish them from the "inner moments" *c*^m^.

To get the same normalization and symmetry properties for the outer and inner form functions, we define the outer form functions  by



It follows





and



The tensors of 3^rd ^order  and of 4^th ^order  own the following symmetry features which are identical for the inner and outer multipole expansion:





We combine the resulting 3, 5 and 7 linearly independent components of the tensors of 2^nd^, 3^rd ^and 4^th ^order to one vector for the marker field  and one vector for the external disturbing field :



The summation of equation (1) and equation (6) yields the field expansion for a magnetic marker with disturbing fields. We truncate this expansion after the octopole terms, and transcript it into a linear equation system for the determination of equivalent multipole moments **c **for a measurement **B**_meas_:



The structure of the vectors **B**_meas _and **c **and the matrix **F **is given below in the formulas (15...24). The residuals 0(·) are sufficiently small, if the coordinates of the marker are small compared to the coordinates of the field sensors , and if the coordinates of the field sensors are small compared to the coordinates of the external disturbing field sources .

**B**_meas _is a vector with the measurement values of the magnetometer sensor field in the positions  with the directions :



The matrix **F **is built from the linearly independent form functions for inner and outer field sources given in equation (13). Their scalar product with the sensor normal directions  yields one row for every sensor:



The number of columns of **F **is the sum of the numbers of inner and outer field functions used. Each column describes the field of one specific magnetic moment with unit strength measured by the sensor system. The Matrix **F **is called the forward matrix of all moments considered. The Matrix **F **is structured into submatrices for different moments:



Matrix  and  are the forward matrices for inner and outer dipoles:



Matrix  and  are the forward matrices for inner and outer quadrupoles. The size of  is (*Nsen*/5) with the rows belonging to quadrupole moments with indices (1,1; 3,3; 1,2; 2,3; 3,1).



Matrix  and  are the forward matrices for inner and outer octopoles. The size of  is (*Nsen*/7) with the rows belonging to octopole moments with indices (1,2,2; 2,3,3; 3,1,1; 1,3,3; 2,1,1; 3,2,2; 1,2,3).



The vector of multipole moments **c **is composed of inner and outer dipole moments **c**_d_, quadrupole moments **c**_q _and octopole moments **c**_o_:



The inner dipole moments  describe a dipole at the point of origin, the outer dipole moments  describe a homogeneous disturbing field:



The  represent a quadrupole at the point of origin. The  describe an external gradient field, whose field strength vanishes at the origin and which has no spatial derivations of 2^nd ^or higher order. This field can be measured by five ideal gradiometers at the origin and can be compared with the creation of software gradiometers.



The  represent an octopole at the origin. The  describe an external gradient field of 2^nd ^order, which has no spatial derivations of 3^rd ^or higher order and whose field strength and spatial derivatives of 1^st ^order vanish at the origin. This field could be measured by 7 ideal second order gradiometers at the origin, it can be compared with the creation of software gradiometers of 2^nd ^order:



Due to its small spatial extension, the magnetic marker can be described as a dipole of strength  at position  as a good approximation. The field of this dipole is



With the Taylor series expansion



follows in analogy to equation (1)



A comparison of coefficients of (1) and (27) yields



and



The dipole strength  can be determined by the dipole moment . An equation system for the adjacent calculation of the dipole position  from the dipole strength  and the quadrupole moment  follows from (29) and (23):



with



This equation system is named shift equation in analogy to [10]. It is overdetermined, and can be solved by means of the pseudo inverse of **m**.



We get the multipole moments **c**, which are required for the localization of the marker dipole, from solving the overdetermined equation system (14) by means of the pseudo inverse of **F**:

**c **= (**F**^T^·**F**)^-1^·**F**^T^·**B**_meas_.     (33)

Here, the matrix of form functions **F **must contain columns at least for the moments  and .

Iterative dipole localization for a fixed dipole (e.g. one time point) is achieved by using the localization position as a new point of origin. The step length of the last localization step serves as a stop criterion for the iterative localization procedure. This is justified by considering the residuals of equation (14) within the convergence range of the procedure, and will practically be shown by the results of the following simulations.

**Figure 1:**
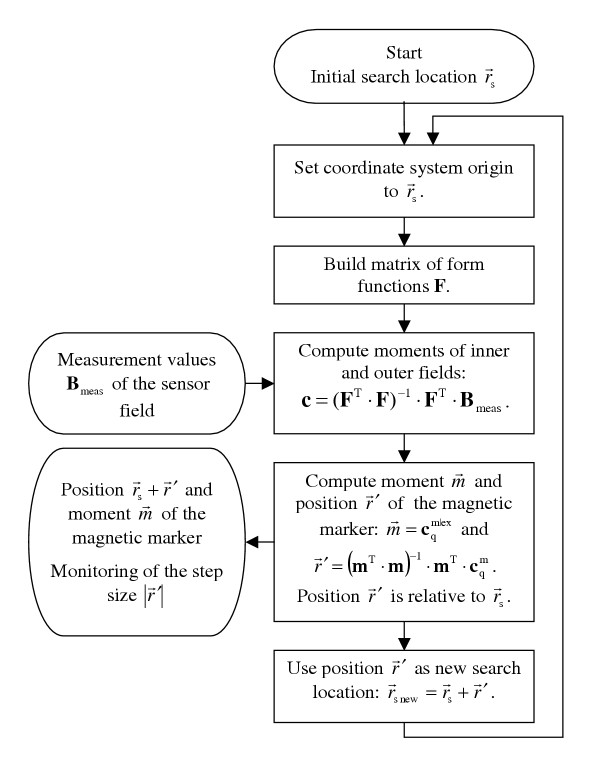
**Flow chart of the algorithm for online localization. **This algorithm is meant for online localization, and therefore comprises only one iteration. A high signal to noise ratio and a high computing speed render 2–3 iterations per measurement cycle possible.

The tracking of a moved dipole based on measurements at consecutive time steps works by updating both the point of origin and the measurement data set after each localization step (Fig. 1). The localization step must be monitored, since it contains information about the marker speed and the noise dependent and speed dependent localization errors.

### Measurement system

**Figure 2:**
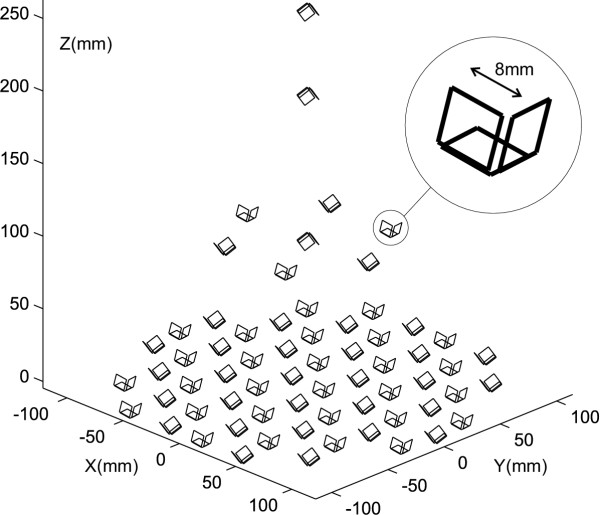
**SQUID Array Argos 200. **The ATB SQUID Array Argos 200 consists of 195 magnetometers which are arranged in orthogonal sensor triplets in four levels. The measurement area of each sensor is a square of 8 mm edge length.

The simulations to determine the performance of the algorithm use the sensor geometry of the multi channel SQUID system Argos 200 from AtB (Advanced Technologies Biomagnetics, Pescara, Italy). The ARGOS 200 system contains fully integrated planar SQUID magnetometers produced using Nb technology with integrated pick-up loops. The sensing area is a square of 8 mm side length. The intrinsic noise level of the built in 195 SQUID sensors is below 5 fT Hz^-1/2 ^at 10 Hz. Three sensors form one orthogonal triplet in each case. The measurement plane with a diameter of 23 cm consists of 56 of those triplets. The reference array consists of seven SQUID sensor triplets located in the second level in a plane which is positioned parallel to the measurement plane at a distance of 98 mm. The third (196 mm above the first plane) and fourth (254 mm above the first plane) levels contain one triplet each (Fig. 2).

The measurement system is positioned within a magnetically shielded room, consisting of 3 highly permeable shieldings and one eddy current shielding. The shielding performance is 38 db at 1 Hz, 55 db at Hz and 80 db at 20 Hz.

The sensor arrangement in orthogonal triplets facilitates the measurement of all 3 spatial components of the magnetic field. Thus, the required field coverage for the localization of a magnetic marker with unknown dipole strength is achieved.

The subdivision into 168 measurement and 27 reference sensors is meant for the creation of software gradiometers. We can use all sensors simultaneously for the multipole method which integrates the suppression of disturbing fields.

With the above described measurement system we performed simulations with different signal to noise ratios.

## Results

We examined the localization characteristics of the multipole method by means of simulation runs at the sensor geometry of the measurement system Argos 200 (Fig. 2). All simulations performed are based on a dipole at position (*x*, *y*, *z*) = (0, 0, -300 mm), i.e. 30 cm below the measurement plane, with a dipole strength of 20 Amm^2^. This is a realistic dipole position for an examination within the digestive tract. The dipole field was superimposed by uncorrelated, Gauss distributed noise. The noise level in fT is also given as signal-to-noise-ratio (SNR), based on the channel with the strongest amplitude of the dipole field.

**Figure 3:**
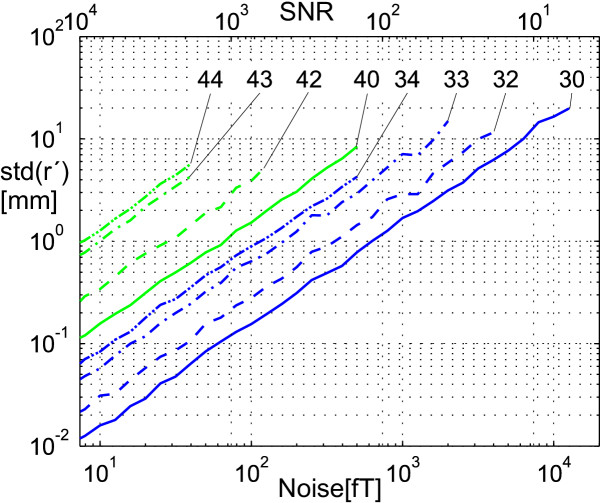
**Noise-dependent localization error. **The mean squared localization error e*rr*() over 100 simulations has been determined depending on the noise level and on the different number of multipole moments used. The curves are plotted up to the noise level, where all simulations still produced a stable localization result. We used the inner moments up to the 3^rd ^order , , plotted in curves 3χ and the inner moments up to the 4^th ^order , , , plotted in curves 4χ. The outer moments which were used to model the disturbing fields were none (curves χ0), 2^nd ^order moments (curves χ2: homogeneous fields), 2^nd ^and 3^rd ^order moments (curves χ3: homogeneous and gradient fields), and 2^nd ^to 4^th ^order moments (curves χ4: external fields up to 2^nd ^order). The simplest disturbing field to model is a homogeneous field having index χ2. The dipole field of a dipole with a strength of 20 Amm^2 ^at position (*x*, *y*, *z*) = (0, 0, -300 mm) is superimposed by white, Gaussian distributed noise, which is given in fT and as the signal to noise ratio (SNR).

The average localization accuracy over 100 simulations has been determined depending on the noise level and on the number of the multipole moments used in the vector **c **(21) (Fig. 3).

The localization was run up to a stable point. We define the localization error as the mean quadratic error of the 100 stable points based on the true dipole position. The localization error increases if we use higher order multipole moments. This holds true for the inner moments **c**^m ^and for the outer moments **c**^ex ^as well. As a good approximation the interrelationship between noise level and localization error is linear, with raising proportionality factor for higher mode numbers. This corresponds to parallel translation of the curves in double logarithmic plotting.

**Figure 4:**
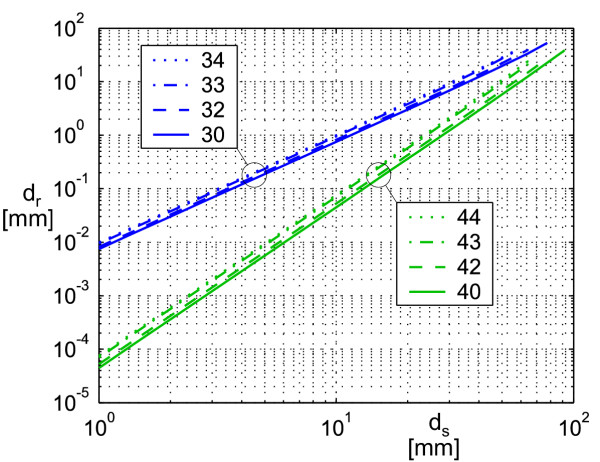
**Localization error depending on the starting point distance for one iteration. **For each distance *d*_*s *_the start position has been moved from the dipole into 100 random directions. The mean remaining distance *d*_*r *_after one localization step is shown. The curves are plotted until the starting distance *d*_*r *_where the localization was still stable from all 100 directions. To get the result after multiple iterative localization steps, the *d*_r_-value has to be taken as the starting distance *d*_*s *_of the following step. We used the inner moments up to the 3^rd ^order , , plotted in curves 3χ and the inner moments up to the 4^th ^order , , , plotted in curves 4χ. The outer moments which were used to model the disturbing fields were none (curves χ0), 2^nd ^order moments (curves χ2: homogeneous fields), 2^nd ^and 3^rd ^order moments (curves χ3: homogeneous and gradient fields), and 2^nd ^to 4^th ^order moments (curves χ4: external fields up to 2^nd ^order).

We examined the localization speed depending on the distance of the starting point to the dipole position. For any tested distance the starting point has been moved from the dipole position into 100 random directions. The remaining mean distance to the dipole position after one localization step is depicted in Fig. 4. The localization speed turns to be significantly higher when using inner octopoles. It gets higher with a shorter starting distance in both an absolute and a relative manner based on the starting distance. Both effects are to be expected directly from the residuals of equation (14). The influence of the outer multipoles on the localization speed is low. The convergence radius at which the dipole was found from all 100 directions decreases slightly with the raising number of outer multipoles used, and increases slightly if inner octopoles are used (unequal right ends of the respective curves in Fig. 4). The convergence radius ranges between 6 and 10 cm. The maximum number of iterations for a target accuracy of 1 mm can be estimated from Fig. 4 as 3.

**Figure 5:**
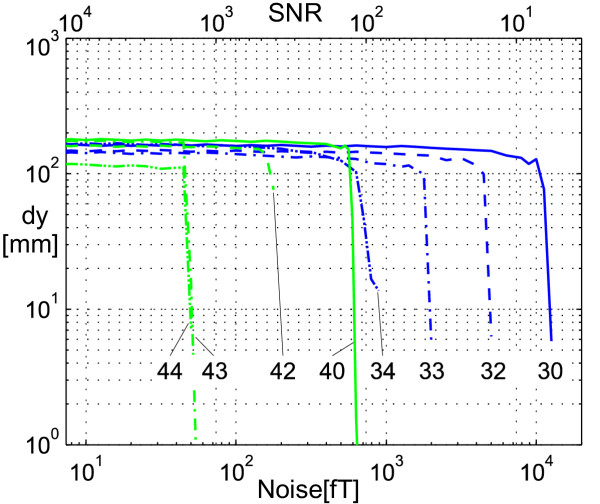
**Convergence distance depending on noise level. **The maximum y-distance *dy *between starting position and dipole position, at which for 100 random noise distributions the dipole could still get localized, is plotted. We used the inner moments up to the 3^rd ^order , , plotted in curves 3χ and the inner moments up to the 4^th ^order , , , plotted in curves 4χ. The outer moments which were used to model the disturbing fields were none (curves χ0), 2^nd ^order moments (curves χ2: homogeneous fields), 2^nd ^and 3^rd ^order moments (curves χ3: homogeneous and gradient fields), and 2^nd ^to 4^th ^order moments (curves χ4: external fields up to 2^nd ^order). The dipole field of a dipole with a strength of 20 Amm^2 ^at position (*x*, *y*, *z*) = (0, 0, -300 mm) is superimposed by white, Gaussian distributed noise given in fT and as the signal to noise ratio (SNR).

In the following we examined the interrelationship between the convergence distance at y-direction and the noise level. The maximum y-distance of the starting position to the dipole, at which the dipole could be found with 100 random noise distributions, is depicted in Fig. 5. It shows that the convergence distance remains unchanged almost up to the point of critical noise level (see Fig. 3) at which localization becomes impossible. The convergence distance, also compare the maximum convergence radius from the curve ends of plot (Fig. 4), depends only marginally on the choice of the inner ansatz functions. It decreases slightly when using outer multipoles. Having a convergence radius of at least 6 cm for the dipole position tested, the choice of the starting position can be regarded as noncritical.

The computing time used for one localization step is 5 ms with an implementation in Matlab at a standard Windows PC with a 2 GHz clock frequency. With maximum 3 iterations per localization step and additional computing time needed for data transfer and a basic visualization, 10 localizations per second are possible. This rate is normally sufficient for marker localizations.

## Discussion

The localization speed rises when using inner octopoles , but this is associated with a higher localization error. At a signal to noise ratio lower than 10^3 ^inner octopoles cannot be used. An SNR of at least 10^2 ^is required for a source positioned 30 cm below the measurement plane.

The outer moments **c**^ex ^used enlarge the localization error depending on the uncorrelated sensor noise, as shown in Fig. 3. Contrary, the localization error depending on the spatially correlated residual field within the measurement room lowers when using outer moments. Depending on the ratio between correlated and uncorrelated noise which has to be found with practical test series, noise suppression of homogeneous disturbing fields using  and possibly noise suppression of gradient fields using  are applicable. To ensure convergence, the starting point for the algorithm has to be within the convergence radius given in Fig. 4. With typical measurement conditions, thus, a starting point 10 cm below the center of the measurement system will suffice.

## Conclusions

The multipole localization is an effective algorithm because it unites a method for the suppression of disturbing fields with a localization method. It can be used iteratively and online for the tracking of magnetic marker timelines within the intestinal tract.
